# Trauma Exposure as a “Driver” of Change in Mental Health Problems Among Youth with Multiple Admissions to Juvenile Detention

**DOI:** 10.3390/ijerph22111710

**Published:** 2025-11-13

**Authors:** Patricia K. Kerig, Jeremiah W. Jaggers, Ava R. Alexander

**Affiliations:** 1 Department of Psychology, University of Utah, Salt Lake City, UT 84112, USA; u1363875@utah.edu; 2 College of Social Work, University of Utah, Salt Lake City, UT 84112, USA; jeremiah.jaggers@utah.edu

**Keywords:** trauma, recidivism, juvenile justice, mental health

## Abstract

Although trauma exposure (TE) has been shown to be a robust predictor of youth involvement in the juvenile justice system, evidence regarding the role of TE amongst youth who recidivate has been more mixed. Recidivist youth are a population of particular concern, given evidence of declining mental health and diminished likelihood of returning to an adaptive developmental course. One way in which TE may contribute to these negative outcomes over time is through potentiating or “driving” mental health problems, which are especially prevalent among trauma-exposed youth in the justice system. To examine this hypothesis, longitudinal data were obtained over a 10-year period from a sample of 5615 juvenile justice-involved youth (1499 girls and 4116 boys) who completed a mental health screening at each admission to detention. Results of analyses assessing the associations among trauma exposure, linear and quadratic time, and mental health problems were consistent with the hypothesis that increases in TE were associated with increasing anger/irritability, depression/anxiety, somatic complaints, and suicidal ideation across repeat admissions. With the exception of alcohol/drug use, all mental health outcomes followed a quadratic trajectory over the course of multiple admissions. Rates of mental health problems were consistently highest for girls and White youth across all waves. These results add to our understanding of the role of trauma in mental health problems among persistent offenders and may help to inform interventions designed to reduce youth contact with the potentially iatrogenic effects of justice system involvement.

## 1. Introduction

A significant body of research confirms that youth in the juvenile justice system have experienced disproportionately high rates of exposure to childhood trauma. Whereas adolescents in community samples typically report rates of trauma exposure (TE) of around 60% [1], studies of youth in juvenile justice settings report TE rates of 90% or higher [2,3,4]. Similarly disproportionate rates are found in clinical samples, in which the subset of youth with juvenile justice contact evince particularly early-onset, frequent, and severe forms of TE [5]. Moreover, research provides compelling evidence that childhood TE is a predictor of adolescent offending and justice system involvement [6,7,8,9,10,11] as well as recidivism [12,13,14,15,16]. Recidivism is an outcome of particular concern given the costs to society—as well as to the youth themselves—of repeat offending. Multiple stays in detention are associated with increasingly negative trajectories for those youth who spend time in the “deep end” [17] of the juvenile justice system, including declining mental health [18,19,20] and diminished likelihood of returning to an adaptive developmental course [21,22,23].

Despite a demonstrated link between TE and recidivism in some studies, McKenna et al. [24] found that the weight of the evidence suggests that TE is associated with initial entry into the justice system but not with persistence. Moreover, other investigations [25] and systematic reviews [26] have not found consistently strong support for the hypothesis that there is a causal association between TE and repeat offending. Consequently, additional research is needed to clarify the association between TE and recidivism as well as the mechanisms that might explain the putative link.

One potential limitation of the existing research is that many relevant studies and conceptual models have focused on posttraumatic stress symptoms (PTSS) as the sole sequelae of TE [27]. Although the major diagnostic manuals concur in identifying PTSS as a key outcome of TE, research shows in fact that posttraumatic stress is only one potential consequence of exposure to trauma [28]. Indeed, childhood TE predicts a wide array of emotional and behavioral problems amongst adolescents, including those on the internalizing spectrum (e.g., anxiety, depression, somatic complaints, and self-harm) and the externalizing spectrum (e.g., anger, irritability, and substance misuse), all of which are seen at disproportionately high rates amongst trauma-exposed youth in the justice system [3,29,30,31]. Therefore, it is important to consider the possibility that TE may be better construed as a transdiagnostic factor [32,33] that acts as a “gateway” to a wide array of mental health problems that contribute to adolescent problem behavior [34].

Another way in which TE may be implicated in mental health problems amongst recidivist youth is through exacerbating potential iatrogenic effects of justice system involvement. A host of studies have shown that deepening levels of involvement in the juvenile justice system are associated with declining mental health, with a clear “dose-response” association between length of time detained and negative outcomes [35]. Youth with a history of childhood TE may have a heightened vulnerability to these effects due to the potential for re-traumatization in secure settings [36], as well as the sensitizing effect of early trauma exposure to future environmental stressors [37,38,39]. Indeed, TE history is associated with exacerbations in aggression and misconduct amongst boys while in secure care [40,41] and TE has been linked to a heightened risk of self-harming behavior amongst girls during system involvement [42]. Thus, both TE and repeated or prolonged incarceration may comprise transdiagnostic risk factors for the development of youth mental health problems. To assess these dynamics, it is important to go beyond studies that take a “snapshot” of youth at one point in time in order to study trajectories of mental health problems over time among youth with persistent involvement in the justice system.

It is also important to consider potential contributions related to youth sex and racial/ethnic identity. Regarding sex, studies consistently find that girls in the justice system report the highest levels of childhood TE, and theoretical models highlight the role of TE in diverting girls onto a pathway toward justice system involvement [43,44,45]. Among the few studies that have looked directly at the role of TE in girls’ recidivism, results have been inconsistent regarding whether this is a gendered phenomenon. For example, Conrad et al. [46] found that, after other risk factors were accounted for, TE in the form of sexual abuse was the strongest predictor of girls’ recidivism but that was not true for boys. In contrast, Vitopolous et al.’s [15] results indicated that TE was an equally strong predictor of recidivism for both boys and girls. Regarding race/ethnicity, minoritized youth are both disproportionately involved in the justice system [47,48] and disproportionately exposed to some forms of TE, such as community violence and race-based traumatic stress [49,50]. Nevertheless, some studies have found that TE plays a differentially strong contributing role in non-Hispanic White youth’s justice involvement [51]. Further, non-Hispanic White youth are generally found to have higher levels of mental health problems overall when compared to youth of color in justice-involved samples [52,53,54]. In sum, given the range of findings in the extant literature, the potential roles of both sex and race/ethnicity require further investigation.

## 2. The Present Study

The purpose of this study was to examine longitudinal trajectories of change in reports of mental health problems, including substance use, anger/irritability, depression/anxiety, somatic complaints, and suicidal ideation, among youth with multiple admissions to detention during a 10-year period, as well as the association between TE and mental health problems at each timepoint. We hypothesized that TE would act as a “driver” of these trajectories and would be associated with increased mental health problems among recidivist youth—those in the “deep end” of the juvenile justice system [17]—over the period during which data were gathered. In addition, potential contributions of youth sex and race/ethnicity were taken into account.

## 3. Method

### 3.1. Participants and Procedure

The present study utilized administrative data from the state of Utah Juvenile Justice Services Administration. Data included all consecutive mental health screenings conducted between 2007 and 2017 at the Salt Lake Detention Center, where young offenders are placed while awaiting case disposition or adjudication, or are serving brief sentences post-adjudication. As part of the standard intake process, within 48 h of admission, youth completed a computer-administered mental health screening measure in English or Spanish, with a detention staff member present in the room but unable to see their responses. The average age at the first timepoint was 15.73 years (SD = 1.40, range from 9.87 to 18.93). As specified in the screening program software, youth self-identified their sex as male or female and their race/ethnicity as one of eight categories: Asian/Asian American, Black, White, Hispanic, Pacific Islander/Native Hawaiian, Native American, Biracial/Multiracial, and Other. Very few youth in the present sample selected the 1st category or the latter 4 categories and therefore those smaller groups were recoded into a single “Other” category, resulting in four total categories: White, Black, Hispanic, and Other. See Table 1 for detailed descriptive statistics.

### 3.2. Measures

The Massachusetts Youth Screening Instrument (MAYSI-2) is a self-report inventory used to screen for mental health-related thoughts, feelings, and behaviors [55]. The instrument comprises 52 yes or no questions, which are scored using the proprietary software MAYSIWARE© (National Youth Screening Assessment Project; Worchester, MA; United States) to create six subscales for girls and seven for boys. The subscales include Alcohol/Drug Use (ADU), Angry-Irritable (AI), Depressed-Anxious (DA), Somatic Complaints (SC), Suicidal Ideation (SI), Thought Disturbance (TD, validated for boys only) and Traumatic Experiences (TE). Youth respond regarding the presence or absence of concerns within the most recent few months on all scales except TE, which enquires about lifetime experiences. The TE subscale also differs from the others in that it includes one item that differs for boys and girls, resulting in gender-specific scoring rubrics. In turn, the TD scale has demonstrated validity only for boys and, therefore, to allow comparisons across gender, TD was excluded from the present analysis.

Overall, the MAYSI-2 has been found to have good psychometric properties, including adequate validity and internal consistency [55,56,57,58]. Although the TE scale did not undergo formal validation in the original studies in which the MAYSI-2 was developed, evidence suggests that TE shows modest correspondence with measures of posttraumatic stress for both boys and girls [59]. Gender differences for MAYSI-2 have been found, with girls consistently demonstrating higher levels of TE as well as mental health problems, particularly anxiety/depression and suicidal ideation [59,60]. Importantly for the present analyses, Jaggers and colleagues [61] found strong evidence of measurement equivalence of the MAYSI-2 across multiple administrations over time.

### 3.3. Data Analysis

Mixed effects modeling was utilized to assess change over time separately for each dependent variable. An unstructured covariance matrix was used. This approach makes no assumptions about correlations among random effects, estimates variance and covariance separately, and is able to handle complex models. All models were calculated using robust standard errors, which provides a method to account for heteroskedasticity, produces more reliable confidence intervals, is preferable for unbalanced data, and accounts for within-cluster correlation [62]. In addition, mixed effects analysis was selected due to its ability to handle large amounts of missing data and for its ability to assess differences within- and between-groups [63]. Only waves one (*n* = 5615) to eight (*n* = 52) were utilized; waves nine to thirteen included only a maximum of *n* = 15 participants and thus were not included in these analyses [64]. Fixed effects, also known as level 1 or within-participant effects, represent the average value over time for each individual. Random effects, also known as level 2 or between-participant effects, represents variability in the outcome that is attributable to variation across individuals.

Dependent variables included ADU, AI, DA, SC, and SI. TE, linear time, and quadratic time were used as the primary predictors in each model. Time was operationalized as the admission number associated with each observation, such that time one refers to the youth’s first admission to detention, time two to the youth’s second admission, and so on. Each dependent variable was recoded with a one-period lag, which permitted modeling of temporal dependencies and exploration of a cause–effect relationship and reduced the impact of serial correlation [65,66]. Simply put, this strategy allows for modeling of the impact of TE on later administrations of the MAYSI-2. Because the TE subscale has a true 0, it was not mean-centered. Quadratic time (time^2^) models curvilinear trends, which includes key turning points [67]. Four models were constructed for each of the dependent variables: Model 1 only included level 1 fixed effects for the dependent variable; Model 2 included fixed effects for TE, linear time, and quadratic time; Model 3 added random effects for time and TE; Model 4 added level one covariates, using Gelman & Hill’s [68] recommended approach, including gender and race/ethnicity. All analyses were conducted using Stata v.18 [69].

Model fit indices included log likelihood, intraclass correlation (ICC), the Akaike Information Criterion (AIC), and the Bayesian Information Criterion (BIC). For model 1, the −2 log likelihood (−2LL) was used. Models 2–4 used the pseudo log likelihood statistic since −2LL could not be calculated due to model complexity. Lower log likelihood, AIC, and BIC values indicate better fit, and were used to make comparisons across each of the four models. ICC was used to quantify the proportion of the total variance that was explained by the level 2 variables [70,71]. In short, ICC explained how strong the relationship was between the dependent variable and between-participant effects.

## 4. Results

**Figure 1:**
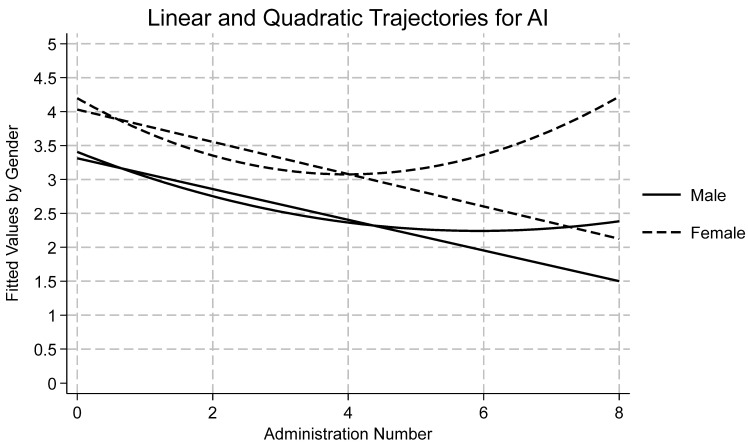
Longitudinal trajectories for anger/irritability (AI).

**Figure 2:**
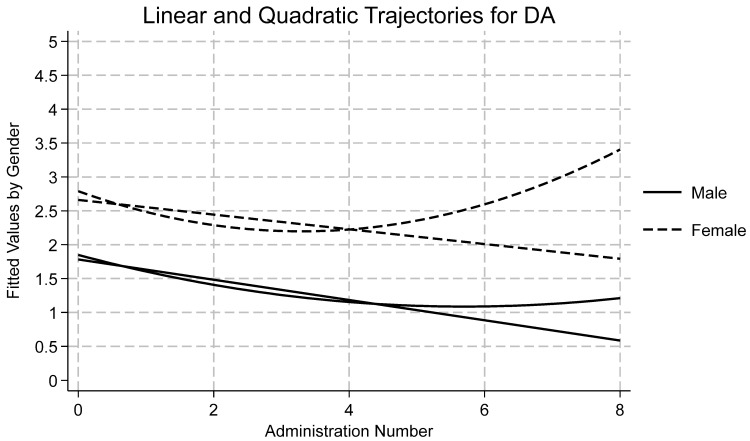
Longitudinal trajectories for depression/anxiety (DA).

**Figure 3:**
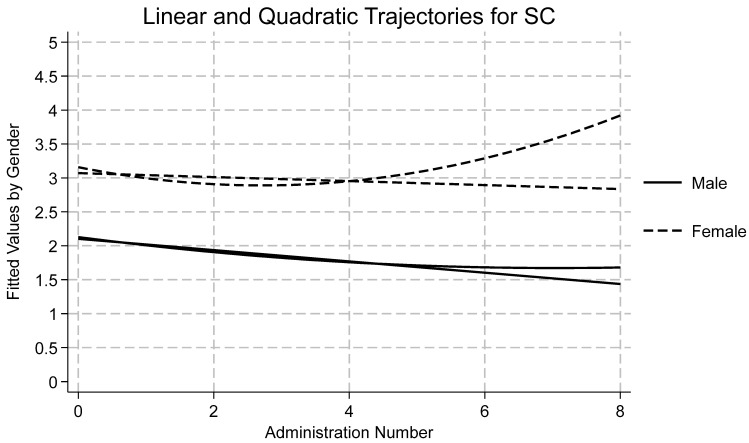
Longitudinal trajectories for somatic complaints (SC).

**Figure 4:**
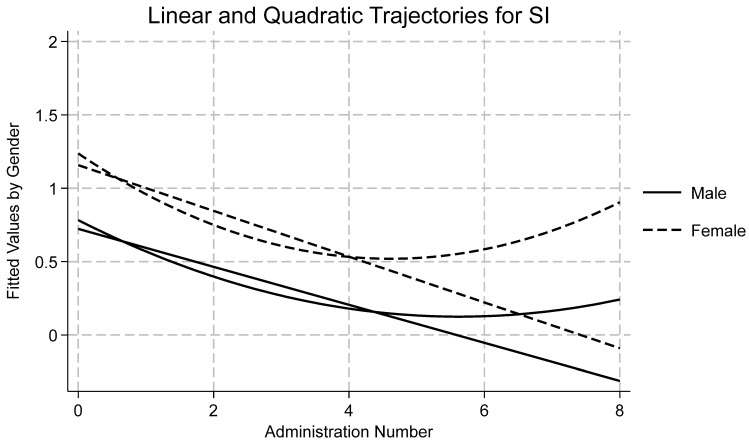
Longitudinal trajectories for suicidal ideation (SI).

Table 1 displays descriptive statistics by time and subscale for all measures as well as reliability statistics for each subscale at the first wave. Skewness and kurtosis were assessed for each variable by timepoint, and all were found to be within accepted limits [72,73]. Using an a priori cut off of ±3.5 standard deviations from the mean, no outliers were detected. Data were missing at random and therefore full information maximum likelihood was used to estimate each model due to its ability to handle missingness. A summary of the findings of each model follows, with emphasis on Model 4 and quadratic time. Results for Model 4 by variable are presented in Table 2. Effects for models one, two, three, and four may be found in the Supplemental Materials (Tables S1–S5). Linear and quadratic trajectories for AI, DA, SC, and SI may be found in Figure 1, Figure 2, Figure 3 and Figure 4, respectively. Linear and quadratic trajectories for ADU may be found in the Supplemental Materials.

### 4.1. Alcohol/Drug Use (ADU)

Using model fit indices, Model 4 was found to have the strongest fit (see Table 2). ICC indicated that nearly 60% of the variation in ADU was attributable to differences between participants. TE was found to be a significant predictor across all four models. On average, individual ADU scores increased by 0.21 for every unit increase in TE. Random effects showed considerable between-participant variation in mean ADU scores, indicating high variability in individual baseline levels. On the other hand, very little change in TE was explained by between-participant differences, meaning there was a largely homogenous relationship between ADU and trauma. Black youth were found to score 1.24 points higher on average than White youth. Similarly, Hispanic youth scored on average 0.43 points lower than White youth. However, there was no effect of time in any model, which suggests that there was no significant systematic change in ADU across time. Consequently, and to preserve space, the tables and figures do not include results for ADU, but these are available in the Supplementary Materials (Figure S1).

### 4.2. Anger/Irritability (AI)

Fit indices showed that Model 4 was found to have the strongest fit (see Table 2). ICC indicated that 57% of the variation in AI was attributable to differences between participants. TE was found to be a significant predictor across all four models. The average longitudinal effect of TE on AI was 0.29, meaning that a one-point increase in TE resulted in a 0.29-point increase in AI, holding all other fixed effects constant. Moreover, linear and quadratic time were significant predictors of change in AI (see Figure 1). Linear time showed an average 0.44 decrease in AI over time. Quadratic time shows a significant, positive coefficient, meaning that initially AI decreased, but as time progressed, AI in fact increased. Among covariates, gender and Hispanic ethnicity were statistically significant. Across time, girls scored 0.53 points higher than boys. Hispanic youth scored 0.58 points lower on the AI scale when compared with White youth. The random effect for AI was 3.89, which was quite large when compared to the fixed average of 3.13, indicating that there was a significant amount of variation across youth in baseline scores. The random effects of TE were minor but indicated some variation between individual youth’s baseline scores. In sum, AI varied considerably over time and between individuals. Similarly, individual change in trauma was noteworthy even though the impact of trauma changed very little between individuals, suggesting youth coming into detention have similar levels of AI.

### 4.3. Depressed/Anxious (DA)

Fit indices showed that Model 4 had the strongest fit (see Table 2). ICC indicated that 37% of the variation in DA is attributable to differences between participants. TE was found to be a significant predictor across all four models. The average longitudinal effect of TE on DA was 0.25, meaning that a one-point increase in TE resulted in a 0.25-point increase in DA. Linear and quadratic time were significant predictors of change in DA (see Figure 2). Linear time showed an average 0.24-point decrease in DA over time. Quadratic time showed a significant, positive coefficient, meaning that initially DA decreased but, as time progressed, DA began to increase. Among covariates, gender and Hispanic ethnicity were statistically significant. Across time, girls scored 0.73 points higher than boys. Hispanic youth scored 0.24 points lower on the DA scale compared with White youth. The random effect for DA was 1.08, which was large when compared to the fixed average of 1.47, indicating a considerable amount of variation across individuals. The random effects of TE and time were negligible but indicated some variation between participants. Overall, there was wide variation in DA scores over time, but scores between participants demonstrated few differences at baseline.

### 4.4. Somatic Complaints (SC)

Fit indices showed that Model 4 had the strongest fit (see Table 2). ICC indicated that 44% of the variation in SC was attributable to differences between participants. TE was found to be a significant predictor across all four models. The average longitudinal effect of TE on SC was 0.18, meaning that a one-point increase in TE resulted in a 0.18-point increase in SC, holding all other fixed effects constant. Moreover, linear and quadratic time were significant predictors of change in SC (see Figure 3). Linear time showed an average 0.14 decrease in SC over time, while quadratic time showed a significant, positive coefficient, meaning that initially SC scores decreased, but as time progressed, SC increased. Among covariates, gender and race/ethnicity were statistically significant. Across timepoints, girls scored nearly one point higher than boys. Black, Hispanic, and Other youth scored 0.6 points lower on the SC scale compared with White youth. The random effect for SC was 1.22, which represented a modest amount of variation across individuals. The random effects of time and TE were minor but indicated some variation between individuals.

### 4.5. Suicidal Ideation (SI)

Initial attempts at modeling SI using an unstructured covariance matrix yielded untrustworthy results as model convergence could not be achieved. Therefore, the more restrictive independent covariance coverage was used instead. This approach differs in that it does not assume covariances between variables are influenced by data dependencies. Fit indices showed that Model 4 had the strongest fit (see Table 2). ICC indicated that 15% of the variation in SI was attributable to differences between participants. TE was found to be a significant predictor across all four models. The average longitudinal effect of TE on SI was 0.09, meaning that a one-point increase in TE resulted in a 0.09-point increase in SI, holding all other fixed effects constant. Moreover, linear and quadratic time were significant predictors of change in SI (see Figure 4). Linear time showed that there was an average 0.22 decrease in SI over time, while quadratic time showed a significant, positive coefficient, meaning that initially SI scores decreased but, as time progressed, SI increased. Among covariates, there were significant effects for gender, and for Hispanic and Other youth. Across time, girls scored 0.34 points higher than boys. Hispanic youth scored 0.2 points lower than White youth whereas youth of other ethnicities scored 0.3 points lower. The random effect for SI was 0.16, which represented a small amount of variation across individuals. The random effect of time was nearly undetectable, meaning that most longitudinal change arose from individual change and not between-group differences. The random effect for TE was minor but indicated some variation between individuals.

## 5. Discussion

This study examined longitudinal trajectories of mental health problems among youth with multiple admissions to detention during a 10-year period, characterized as those in the “deep end” [17] of the juvenile justice system. Further, we tested trauma-informed theories of the link between TE and subsequent shifts in mental health problems amongst justice-involved youth, positing that TE would act as a potentiator or “driver” of mental health problems over time. We also attended to potential contributions of gender and racial/ethnic identity to these results.

The analyses revealed results that were largely consistent with the study’s hypotheses. With the exception of ADU, which did not systematically change across time, the trajectories of all other mental health problems indicated an initial decrease which was followed by an overall increase across repeat admissions to detention. These patterns are consistent with the idea of iatrogenic effects associated with deepening levels of involvement in the juvenile justice system [23,35,47]. However, these results also may reflect reciprocal effects, in that youth whose mental health needs are not addressed either while in custody or during periods of release into the community—which is all too common [74,75,76]—are increasingly likely over time to persist in problem behavior that brings them into conflict with legal authorities.

Examination of covariates also revealed consistent gender differences, with girls scoring higher that boys across timepoints on measures of AI, AD, SC, and SI. These results are consistent with those of other studies showing disproportionately high mental health needs amongst girls in the justice system [4,60,77]. There also were overall differences related to race/ethnicity, with White youth scoring higher than Hispanic youth across time periods on AI, DA, and SI, and higher than both Hispanic and Black youth on SC. These results also concur with those of other studies in which White youth in detention settings are characterized by the highest levels of emotional and behavioral problems [17,52,54]. One possible explanation, as Winkelman et al. [78] propose, is that for youth of color, structural risk factors, such as socioeconomic disadvantage and racial discrimination, may be a larger driver of justice involvement than mental health problems.

In turn, although due caution must be exercised before making presumptions of causality, the results of these analyses were consistent with the hypothesis that TE may act as a “driver” of changes in mental health functioning across multiple admissions to detention. Across all subscales of the MAYSI-2, higher TE scores were found to predict subsequent increases in mental health problems in models controlling for admission number and demographics, with effects ranging from 0.09 for SI to 0.29 for AI. In a broader sense, these results are consistent with developmental psychopathology models of trauma that posit TE to serve as a transdiagnostic mechanism that potentiates a series of negative developmental cascades [79] which increasingly entrain youth on a maladaptive developmental pathway and reduce the likelihood of resilience [80,81].

The finding that there was no systematic change over time on the MAYSI-2 ADU suggests that fluctuations in substance use may be driven primarily by shifts in youths’ environment or mindset rather than by underlying growth processes. Although TE was found to be a significant predictor of ADU across timepoints, substance use may be affected by other factors, such as the proportion of time youth have spent in secure settings where illicit substances are difficult to procure. Alternatively, the lack of systematic change in ADU over consecutive admissions might reflect the fact that this scale is unique in inquiring about behaviors that represent not only mental health problems but also offenses for which youth might receive further sanctions. Consequently, youth might be motivated to minimize reporting of any illicit substance use during screenings over the course of their trajectories of justice system involvement. Of course, it is a limitation of all self-report measures that they are subject to vagaries in youths’ willingness to disclose, and this is a particularly relevant concern in juvenile justice contexts. Youth who have had negative experiences with the legal system, or whose attitudes toward socialization agents are characterized by legal cynicism, may be particularly unwilling to provide candid responses during detention admissions [36]. Further, although youth reports on mental health screenings are protected against self-incrimination in some jurisdictions, this is not always the case [82], and thus youth concerns about the consequences of such disclosures may be quite valid [83]. Nevertheless, substance abuse problems are highly prevalent amongst youth in the justice system and are also associated with a heightened risk of recidivism [84], and so their detection is a high priority during mental health screenings.

The finding that the longitudinal associations between TE and SI were weaker than those for other mental health problems was unexpected. Although the robustness of this finding will require replication in other samples, one possible explanation is that, similar to avoiding the sanctions that might be associated with acknowledging ADU, over the course of multiple experiences with detention, youth may come to recognize that reporting SI is associated with unwanted consequences that are particularly aversive to trauma-exposed youth, such as being placed on suicide watch and scrutinized closely by staff, isolated from peers, required to wear “scrubs”, or deprived of other liberties.

There are potentially valuable clinical and policy implications of these results. The findings underscore the importance of identifying youth with mental health needs and providing relevant care early in their initial contact with the juvenile justice system, ideally with a preventative lens aimed at interrupting negative patterns of declining mental health and persistent offending. Interventions have been found to effectively divert youth from an antisocial pathway [47], particularly when they are targeted to dynamic risk factors that are modifiable, such as mental health problems [85]. Trauma-informed care in particular is coming to play an important role in both practice and policy in the juvenile justice arena [86,87] and is associated with beneficial effects [36,88]. Another policy consideration is suggested by the possibility that youth responses on the MAYSI-2 might be affected by fears of negative sanctions, such as in the case of disclosures of ADU, which underscores the importance of developing juvenile justice system intake procedures that clarify for youth their rights, the bounds of confidentiality, and the uses to which their reports on mental health screenings will be put [36,83,89]. The fact that legal statutes designed to protect youth from self-incrimination during mental health processing vary widely across jurisdictions also speaks to a need to revisit this concern at the level of national policy [90].

Overall, this study is characterized by several strengths. First, the dataset utilized was relatively large and included all sequential admissions to detention over a 10-year period; consequently, there were no selection biases. The inclusion of girls also represents a valuable contribution to the literature, given that many studies of the associations between mental health problems and recidivism have been conducted on male-only samples (e.g., [91]). In addition, studies investigating potential iatrogenic effects of detention sometimes have followed youth over relatively brief periods of time, which might not have been sufficient to allow effects on mental health functioning to emerge. This study took a step toward redressing that gap by identifying youth in the “deep end” of the justice system, who were characterized by a persistent course of recidivism involving multiple detentions within a 10-year period.

There also are a number of limitations that should also be taken into consideration when interpreting these results. These data were all gathered from a specific geographic region in which the representation of ethnic/racial diversity differs from that in other areas of the United States. In particular, although the composition of the sample was consistent with that of the larger community from which the data were drawn, relatively few Black youth were represented in this sample. Further research is needed that includes samples that are more diverse and representative of the larger US population. The MAYSI-2 also provides only a limited assessment of youth identities, such as by not distinguishing between race and ethnicity. In future research, it also will be valuable to investigate intersectionality, in that there may be ways in which gender and race/ethnicity interact to predict outcomes related to the links among TE, mental health problems, and recidivism [92].

A further limitation of the system-derived data utilized in the present study is that they did not provide information regarding the specific offenses youth committed at each timepoint. In some jurisdictions, youth are returned to detention due to technical violations rather than new offenses and are thus not true recidivists; however, this is not likely in the current context, given local diversion-oriented sentencing guidelines which preclude placing youth in detention for mere technical violations. These data also leave open the question as to why some youth did not return to detention: whereas some may have desisted in offending, others may have relocated to a different jurisdiction or been placed in long-term correctional institutions. Similarly, data were not available regarding adult criminal system involvement for youth who aged out of the juvenile system during the time period covered by this study.

Further, information was not available regarding any services youth might have received during the timespan of the study, including mental health interventions, which might have affected their trajectories. Nevertheless, although the provision of mental health services has been associated with a reduced likelihood of reoffending among some subsets of young offenders [75,93], studies on large samples show that only a small number of those justice-involved youth who are in need are referred to mental health services while they are in custody [75,76] and even fewer retain access to services upon their release into the community [74]. Consequently, rates of mental health problems remain high among detained youth long after their release [94]. On the other hand, given that longitudinal studies also show that improvements in mental health are associated with reduced youth offending [95], provision of such services promises to benefit the justice system and society by reducing recidivism.

Yet another limitation of this study is that the screening measure utilized did not include a measure of PTSS. Although the TE scale is modestly associated with validated measures of PTSS [59], it primarily assesses exposure to traumatic experiences rather than posttraumatic symptoms per se. Even though the other mental health problems assessed in this study are highly comorbid with PTSS [83] and are frequently observed sequela of TE [28], PTSS likely plays a contributing role in youth recidivism, including potentially sensitizing youth to the iatrogenic effects of system involvement [36]. In addition, consistent with other studies investigating gender differences on the MAYSI-2, the TD scale was not included in these analyses due to its lack of established validity with girls. However, research has shown that TE is associated with thought disorder and other psychotic symptoms amongst youth [96,97], and these are prevalent mental health problems in justice-involved samples [29]. Given the profound negative implications of psychosis for youth functioning, and the importance of early intervention in psychotic processes, the need is high for screening instruments that are valid for girls.

These data also did not allow us to identify the source(s) of TE, which for some youth might have been related to justice system contacts or the detention experience itself [23,49]. In this way, the potential iatrogenic effects of juvenile justice involvement also might take the form of new trauma exposures. Justice-involved youth report high rates of many types of TE while in detention, including witnessing and being victimized by physical and sexual violence, restraints, and other harsh punitive measures [98,99]. For example, in one large-scale survey of over 8500 detained youth, Ahlin [100] found that approximately 10 percent reported having experienced a sexual violation while in custody. The clinical literature suggests that these experiences may be particularly distressing for youth with prior histories of TE [36,101]. Moreover, Dierkhising et al.’s [98] cross-sectional study found that experiencing abuse while in secure care was correlated not only with PTSD and depression, but with elevated rates of recidivism. Accordingly, the contribution of justice system-related trauma on trajectories of youth’s mental health functioning and recidivism will be a valuable direction for further longitudinal research.

Additionally, for the purposes of this study, time was operationalized as the admission number associated with the MAYSI administration for each youth. However, it should be noted that admissions were not evenly spaced in terms of time or youth age. Accordingly, this study examines underlying growth processes related to deepening involvement in the juvenile justice system but is not able to account for other simultaneous or overlapping developmental processes, such as increasing maturity. Finally, it should be noted that the first observation available for each youth might not have been that youth’s first experience with the juvenile justice system and thus there may have been previous admissions prior to the timeframe of this study; similarly, youth may have engaged in further offending after the time period of this study ended. Accordingly, a valuable step for future research would be to start with a sample of first-time offenders and to follow them throughout their trajectories from adolescence into adulthood.

## 6. Conclusions

In conclusion, this study adds to the growing body of research investigating the role of childhood trauma in the trajectories of youth both “to” and “through” the juvenile justice system [24]. Recidivist youth with histories of TE are vulnerable to declining emotional and behavioral health across multiple admissions to detention. These mental health problems in turn may contribute not only to functional impairments but to a persistent negative developmental course, underscoring the value of taking a trauma-informed approach to prevention and intervention in juvenile justice.

## Figures and Tables

**Table 1:** Descriptive statistics.

	ADU Mean (SE)	AI Mean (SE)	DA Mean (SE)	SC Mean (SE)	SI Mean (SE)	TE Mean (SE)
TIME 1 (*n* = 5615)	2.29 (0.03)	2.99 (0.04)	1.85 (0.03)	2.32 (0.02)	0.67 (0.02)	1.84 (0.02)
TIME 2 (*n* = 2289)	2.52 (0.05)	2.67 (0.05)	1.57 (0.04)	2.10 (0.04)	0.41 (0.02)	1.69 (0.03)
TIME 3 (*n* = 1255)	2.67 (0.07)	2.63 (0.07)	1.44 (0.05)	2.15 (0.05)	0.33 (0.03)	1.61 (0.04)
TIME 4 (*n* = 709)	2.76 (0.10)	2.52 (0.10)	1.38 (0.07)	2.04 (0.07)	0.29 (0.03)	1.58 (0.06)
TIME 5 (*n* = 392)	2.76 (0.13)	2.33 (0.13)	1.42 (0.09)	1.94 (0.10)	0.26 (0.04)	1.46 (0.08)
TIME 6 (*n* = 210)	2.95 (0.18)	2.45 (0.17)	1.38 (0.12)	1.95 (0.14)	0.18 (0.05)	1.46 (0.10)
TIME 7 (*n* = 112)	2.71 (0.24)	2.09 (0.21)	1.26 (0.15)	1.92 (0.19)	0.21 (0.06)	1.46 (0.14)
TIME 8 (*n* = 52)	2.81 (0.36)	2.04 (0.28)	1.02 (0.19)	2.19 (0.31)	0.13 (0.08)	1.33 (0.21)
	**N (%)**		**Cronbach’s Alpha**			
Female	1499 (26.7%)		ADU	0.81		
Male	4116 (73.3%)		AI	0.82		
White	2826 (50.3%		DA	0.72		
Black	334 (5.9%)		SC	0.73		
Hispanic	1955 (34.8%)		SI	0.86		
Other	500 (8.9%)		TE-girls	0.69		
			TE-boys	0.61		

**Table 2:** Model 4 results.

	Anger/Irritability	Depression/Anxiety	Somatic Complaints	Suicidal Ideation
FIXED EFFECTS				
Intercept	3.13 (0.13) *	1.47 (0.09) *	2.13 (0.09) *	0.71 (0.06) *
Time	−0.44 (0.08) *	−0.24 (0.05) *	−0.14 (0.05) *	−0.22 (0.03) *
Time^2^	0.05 (0.01) *	0.03 (0.01) *	0.02 (0.01) *	0.02 (0.01) *
Trauma	0.29 (0.03) *	0.25 (0.02) *	0.18 (0.02) *	0.09 (0.01) *
Gender (female)	0.53 (0.11) *	0.73 (0.08) *	0.9 (0.08) *	0.34 (0.05) *
Black	−0.13 (0.24)	−0.06 (0.13)	−0.63 (0.14) *	−0.10 (0.08)
Hispanic	−0.58 (0.1) *	−0.24 (0.07) *	−0.6 (0.07) *	−0.2 (0.04) *
Other	0.01 (0.17)	−0.25 (0.1) *	−0.6 (0.11) *	−0.3 (0.06) *
RANDOM EFFECTS				
Participant	3.89 (0.39)	1.08 (0.23)	1.22 (0.02)	0.16 (0.01)
Time	0.21 (0.04)	0.04 (0.02)	0.05 (0.02)	<0.001 (<0.001)
Trauma	0.09 (0.04)	0.09 (0.02)	0.05 (0.02)	0.06 (0.01)
FIT				
Log likelihood	−11,340.47	−9675.68	−9527.07	−7551.84
ICC	0.57 (0.03)	0.37 (0.06)	0.44	0.15 (0.06)
AIC	22,710.94	19,381.36	19,084.11	15,127.69
BIC	22,808.80	19,479.22	1981.97	15,205.97

Note: * *p* < 0.05. ICC conditional on zero values for random-effects covariates.

## Data Availability

Data are unavailable due to privacy restrictions.

## Supplementary Materials

The following supporting information can be downloaded at https://www.mdpi.com/article/10.3390/ijerph22111710/s1: Figure S1: Longitudinal Trajectories for ADU; Table S1: Effects for Alcohol/Drug Use; Table S2: Effects for Anger/Irritability; Table S3: Effects for Depression/Anxiety; Table S4: Effects for Somatic Complaints; Table S5: Effects for Suicidal Ideation.
