# Supplementary material for: Trauma Exposure as a “Driver” of Change in Mental Health Problems Among Youth with Multiple Admissions to Juvenile Detention
Source: Int J Environ Res Public Health. 2025 Nov 13;22(11):1710. doi: 10.3390/ijerph22111710 (PMC12652392; doi:10.3390/ijerph22111710)
Supplement: Supplementary file 1 [file ijerph-22-01710-s001.zip › ijerph-3833941-supplementary.pdf]

Figure S1: Longitudinal Trajectories for ADU

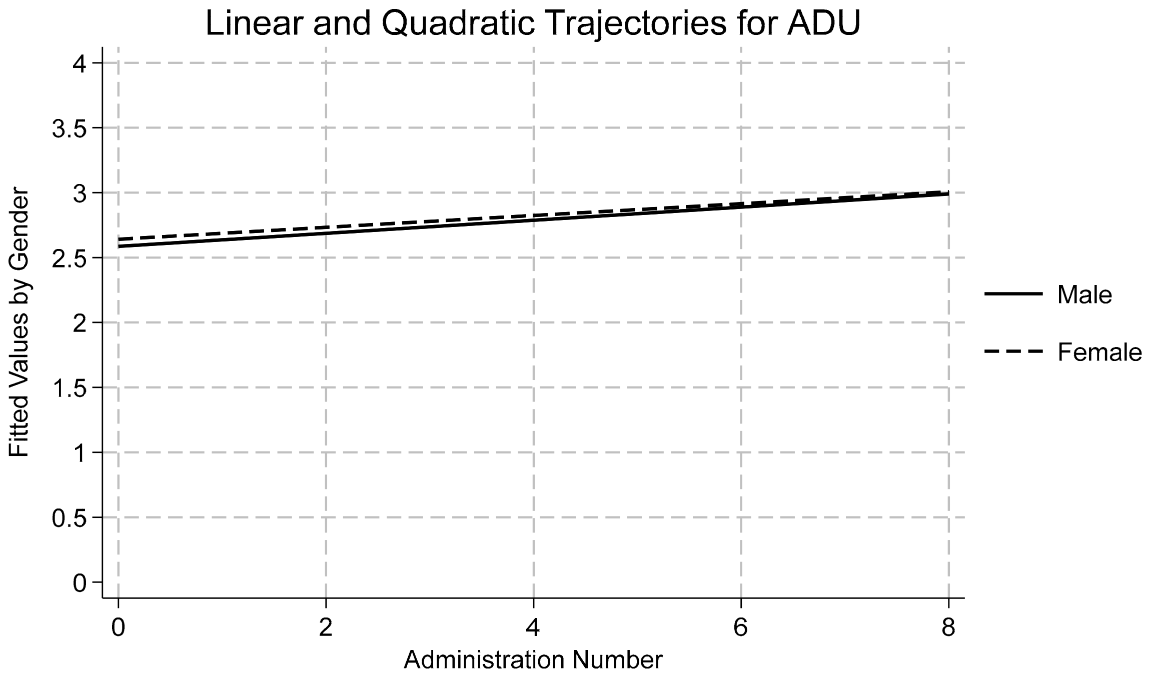

Table S1: Effects for Alcohol/Drug Use

	Alcohol/Drug Use			
**FIXED EFFECTS**	Model 1	Model 2	Model 3	Model 4
Intercept	2.32(0.03)*	2.16(0.1)*	2.18(0.1)*	2.44(0.12)*
Time	-	0.05(0.08)	0.05(0.08)	0.04(0.08)
$Time^2$	-	<0.001(0.01)	<0.001(0.01)	0.001(0.01)
Trauma	-	0.24(0.02)*	0.22(0.02)*	0.21(0.02)*
Gender (female)	-	-	-	-0.09(0.11)
Black	-	-	-	1.24(0.17)*
Hispanic	-	-	-	-0.43(0.1)*
Other	-	-	-	0.06(0.18)
**RANDOM EFFECTS**				
Subject	3.21(0.1)	3.09(0.14)	3.63(0.31)	3.49(0.31)
Time	-	-	0.13(0.03)	0.13(0.03)
Trauma	-	-	0.04(0.03)	0.04(0.03)
**FIT**				
Log likelihood	-23468.32	-11022.8	-10991.06	-10964.41
ICC	0.54(0.01)	0.53(0.02)	0.6(0.03)	0.59(0.03)
AIC	46942.63	22057.60	22004.12	21958.81
BIC	46964.45	22096.74	22075.89	22056.67

*p<0.05

Table S2: Effects for Anger/Irritability

	Anger/Irritability			
**FIXED EFFECTS**	Model 1	Model 2	Model 3	Model 4
Intercept	2.82(0.03)*	2.94(0.11)*	2.99(0.11)*	3.13(0.13)*
Time	-	-0.41(0.08)*	-0.44(0.08)*	-0.44(0.08)*
Time^2	-	0.04(0.01)*	0.05(0.12)*	0.05(0.01)*
Trauma	-	0.34(0.03)*	0.32(0.03)*	0.29(0.03)*
Gender (female)	-	-	-	0.53(0.11)*
Black	-	-	-	-0.13(0.24)
Hispanic	-	-	-	-0.58(0.1)*
Other	-	-	-	0.01(0.17)
**RANDOM EFFECTS**				
Subject	3.58(0.12)	3.04(0.16)	4.09(0.39)	3.89(0.39)
Time	-	-	0.2(0.04)	0.21(0.04)
Trauma	-	-	0.1(0.04)	0.09(0.04)
**FIT**				
Log likelihood	-24455.39	-11410.84	-11370.43	-11340.47
ICC	0.51(0.01)	0.47(0.02)	0.58(0.03)	0.57(0.03)
AIC	48916.80	22833.67	22762.86	22710.94
BIC	48938.61	22872.82	22834.62	22808.80

*p<0.05

Table S3: Effects for Depression/Anxiety

	Depression/Anxiety			
**FIXED EFFECTS**	Model 1	Model 2	Model 3	Model 4
Intercept	1.75(0.02)*	1.49(0.08)*	1.49(0.08)*	1.47(0.09)*
Time	-	-0.23(0.05)*	-0.24(0.05)*	-0.24(0.05)*
Time^2	-	0.03(0.01)*	0.03(0.01)*	0.03(0.01)*
Trauma	-	0.29(0.02)*	0.28(0.02)*	0.25(0.02)*
Gender (female)	-	-	-	0.73(0.08)*
Black	-	-	-	-0.06(0.13)
Hispanic	-	-	-	-0.24(0.07)*
Other	-	-	-	-0.25(0.1)*
**RANDOM EFFECTS**				
Subject	2(0.07)	1.24(0.11)	1.12(0.24)	1.08(0.23)
Time	-	-	0.04(0.02)	0.04(0.02)
Trauma	-	-	0.08(0.02)	0.09(0.02)
**FIT**				
Log likelihood	-21433.23	-9802.09	-9732.02	-9675.68
ICC	0.51(0.01)	0.38(0.03)	0.38(0.06)	0.37(0.06)
AIC	42872.46	19616.18	19486.04	19381.36
BIC	42894.28	19655.32	19557.80	19479.22

*p<0.05

Table S4: Effects for Somatic Complaints

	Somatic Complaints			
**FIXED EFFECTS**	Model 1	Model 2	Model 3	Model 4
Intercept	2.27(0.02)*	1.94(0.08)*	1.97(0.07)*	2.13(0.09)*
Time	-	-0.12(0.06)*	-0.14(0.05)*	-0.14(0.05)*
$Time^2$	-	0.02(0.01)	0.02(0.01)*	0.02(0.01)*
Trauma	-	0.23(0.02)*	0.22(0.02)*	0.18(0.02)*
Gender (female)	-	-	-	0.9(0.08)*
Black	-	-	-	-0.63(0.14)*
Hispanic	-	-	-	-0.6(0.07)*
Other	-	-	-	-0.6(0.11)*
**RANDOM EFFECTS**				
Subject	1.88(0.06)	1.53(0.08)	1.42(0.17)	1.22(0.02)
Time	-	-	0.05(0.02)	0.05(0.02)
Trauma	-	-	0.06(0.02)	0.05(0.02)
**FIT**				
Log likelihood	-20868.83	-9665.95	-9639.68	-9527.07
ICC	0.52	0.47	0.47	0.44
AIC	41743.65	19343.90	19301.36	19084.11
BIC	41765.47	19383.04	19373.12	1981.97

*p<0.05

Table S5: Effects for Suicidal Ideation

	Suicidal Ideation			
**FIXED EFFECTS**	Model 1	Model 2	Model 3	Model 4
Intercept	0.56(0.01)*	0.69(0.05)*	0.65(0.05)*	0.71(0.06)*
Time	-	-0.24(0.03)*	-0.22(0.03)*	-0.22(0.03)*
$Time^2$	-	0.02(0.004)*	0.02(0.01)*	0.02(0.01)*
Trauma	-	0.11(0.01)*	0.11(0.01)*	0.09(0.01)*
Gender (female)	-	-	-	0.34(0.05)*
Black	-	-	-	-0.10(0.08)
Hispanic	-	-	-	-0.2(0.04)*
Other	-	-	-	-0.3(0.06)*
**RANDOM EFFECTS**				
Subject	0.57(0.03)	0.43(0.06)	0.17(0.07)	0.16(0.01)
Time	-	-	<0.001(<0.001)	<0.001(<0.001)
Trauma	-	-	0.06(0.01)	0.06(0.01)
**FIT**				
Log likelihood	-16600.33	-7671.12	-7596.24	-7551.84
ICC	0.39	0.32(0.04)	0.16(0.07)	0.15(0.06)
AIC	33206.66	15354.24	15208.48	15127.69
BIC	33228.48	15393.39	15260.68	15205.97

*p<0.05
